# Successful Management of a Massive Second‐Trimester Subchorionic Hematoma: A Case Report

**DOI:** 10.1155/carm/6154618

**Published:** 2026-08-31

**Authors:** Jianfa Wu, Huang Chen, Qianyi Liao, Yanmei Zhu, Yao Jiang, Zhou Liu

**Affiliations:** ^1^ Department of Gynecology, Affiliated Zhoupu Hospital, Shanghai University of Medicine & Health Sciences, Shanghai, China, sumhs.edu.cn; ^2^ Department of Gynecology, Shanghai University of Medicine & Health Sciences, Shanghai, China, sumhs.edu.cn; ^3^ Department of Integrated Traditional Chinese and Western Medicine Clinical Practice, Shanghai University of Traditional Chinese, Medicine, Shanghai 200032, China; ^4^ Department of Gynecology, Gongshan People’s Hospital, Nujiang Lisu Autonomous Prefecture, China

**Keywords:** case report, massive subchorionic hematoma, miscarriage, second trimester, treatment

## Abstract

A massive subchorionic hematoma is associated with an increased risk of adverse pregnancy outcomes, including spontaneous abortion, preterm birth, and intrauterine fetal demise. However, current clinical guidelines lack standardized diagnostic criteria and evidence‐based management recommendations for massive subchorionic hematomas, particularly during the second trimester. A 34‐year‐old pregnant woman at 12 weeks and 6 days’ gestation presented with acute‐onset vaginal bleeding of 1‐h duration. Color Doppler ultrasonography revealed an anteriorly located placenta and identified a hypoechoic, well‐defined subchorionic collection measuring 109 × 41^2^ mm beneath the chorionic plate of the gestational sac, which was considered a massive subchorionic hematoma at the second trimester. Following written informed consent, intramuscular progesterone was administered to suppress uterine activity, and intravenous cefuroxime was initiated for prophylaxis against infection. Serial monitoring of maternal hemoglobin concentration and subchorionic hematoma dimensions was performed. Following more than 3 months of active fetal surveillance and supportive obstetric management, the patient remained clinically stable. The subchorionic hematoma resolved completely by 28 weeks and 3 days’ gestation, and a viable female neonate was delivered by cesarean section at 39 weeks and 1 day’s gestation. In conclusion, this study provides a detailed, real‐world example of managing a high‐risk, massive subchorionic hematoma in the absence of standardized guidelines, which lays a practical foundation for future research.

## 1. Introduction

Massive subchorionic hematoma (SCH) is associated with an increased risk of secondary fetal growth restriction and stillbirth [1–3]. Consequently, its clinical management remains challenging, and evidence‐based treatment and surveillance guidelines are currently lacking.

This case report describes a patient with a massive SCH diagnosed in the second trimester, who achieved favorable maternal and fetal outcomes following conservative management. These findings challenge the historically cautious prognostic outlook associated with this condition, which purportedly associated with elevated risks of intrauterine fetal demise and severe fetal growth restriction. The principal clinical significance of this case resides in its atypical etiology: the patient had a singleton pregnancy without identifiable conventional risk factors, including chronic hypertension, multifetal gestation, or prior assisted reproductive technology use. The spontaneous development of a massive SCH in the absence of these established predisposing factors underscores the need for further investigation into the underlying pathophysiological mechanisms.

From a therapeutic perspective, this case contributes robust real‐world evidence to a domain lacking consensus‐based clinical guidelines. In response to a massive SCH identified at 12 weeks and 6 days’ gestation, a multidisciplinary team implemented a structured conservative management protocol comprising intramuscular progesterone administration to attenuate uterine activity, intravenous cefuroxime for infection prophylaxis, and intensive surveillance, including serial measurement of maternal serum biomarkers and scheduled transabdominal ultrasonography. Finally, the parturient underwent a term cesarean section and delivered a live neonate, with no other complications occurring.

This report aims to contribute a detailed clinical reference to the existing literature on the management of massive SCH in the absence of standardized guidelines.

## 2. Case Presentation

**FIGURE 1:**
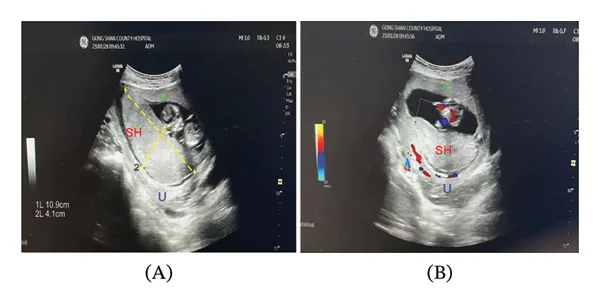
Color Doppler ultrasound images of the fetus and placenta at 12 weeks plus 6 days of gestation. (A) The fetus and placenta were observed by LOGIQ S8 (GE, USA) using a 5.0‐Hz transducer. It was revealed that the placenta was implanted on the anterior uterine wall. A well‐demarcated, hypoechoic zone measuring 109 × 41 mm^2^, representing the largest cross‐sectional area, was identified beneath the gestational sac chorionic plate. Fetal examination is normal. The two yellow dashed lines are the measurement lines for subchorionic hematoma. (B) The blood flow of subchorionic hematoma was also observed with LOGIQ S8. No discernible intraparenchymal or perilesional vascularity was observed within the hematoma region. P represents the placenta; SH represents the subchorionic hematoma; U represents the uterus.

A 34‐year‐old woman at 12 weeks and 6 days’ gestation presented with acute‐onset vaginal bleeding of 1‐h duration. Color Doppler ultrasonography demonstrated anterior placental implantation and revealed a well‐demarcated, hypoechoic subchorionic collection measuring 109 × 41 mm^2^, corresponding to its maximal cross‐sectional area, located beneath the chorionic plate of the gestational sac. No discernible intraparenchymal or perilesional vascularity was observed within this region (Figure 1A,B). There was no obvious abdominal tenderness on physical examination. The patient conceived naturally and had a history of one previous cesarean section for twin pregnancy, a history of 1 stillbirth in the third trimester, and 1 stillbirth at term. The patient had no history of chronic hypertension, pregestational diabetes mellitus, chronic kidney disease, autoimmune disease, pelvic inflammatory disease, abdominal trauma, or systemic vasculopathy. She was not receiving long‐term anticoagulation therapy and reported no medication use other than those prescribed during this pregnancy; no drug allergies were documented.

In this case, the patient’s ultrasonography indicated that the hematoma was located beneath the gestational sac chorionic plate. Combined with the absence of acute abdominal pain, a low‐echo area behind the placenta, and manifestations of multiple cystic dilatation or vascular malformation, diseases including placental abruption, retroplacental hematoma, and placental mesenchymal dysplasia could be excluded, and a diagnosis of SCH was established.

Given the potentially complications associated with pregnancy continuation, including severe hemorrhage, miscarriage, stillbirth, placental abruption, and premature delivery, the patient underwent comprehensive, documented risk‐benefit counseling and provided written informed consent prior to initiating inpatient expectant management. Despite full understanding of the significant maternal risks, she unequivocally affirmed her intention to pursue fetal preservation. She articulated this commitment with clarity and resolve: “This is a long‐awaited pregnancy; I am willing to accept the associated risks in pursuit of a viable outcome.” Her decision was consistently supported by her mother and extended family, all of whom explicitly affirmed their endorsement of her refusal of pregnancy termination. Grounded in the ethical foundations of respect for autonomy and collaborative decision‐making, a multidisciplinary clinical team designed and implemented a conservative, individualized management strategy intended to concurrently safeguard maternal hemodynamic stability and optimize fetal viability. The strategy comprised: (1) intramuscular injection of progesterone (20 mg) once daily was administered for 1 consecutive week to maintain uterine quiescence and sustain ongoing pregnancy [4, 5]; (2) prophylactic intravenous administration of cefuroxime (1.5 g, twice daily) was performed for 48 h to reduce the risk of secondary infection of the hematoma [6]; and (3) protocol‐adherent surveillance to assess hemoglobin trends and retroplacental hematoma dynamics, including serial laboratory tests and targeted transabdominal ultrasound. Surveillance outcomes are reported in Tables 1, 2, 3, and 4.

**TABLE 1:** Timeline and results of laboratory tests (Blood routine analysis).

Test time	Gestational age (weeks)	Test item	Test result	Reference value
2023–1–28	12 weeks and 6 days	Routine analysis of blood	Total white blood cells: 8.60 × 10^9^/L	4–10 × 10^9^/L
			Hemoglobin: 126 g/L	110–160 g/L
			Blood platelet: 247 × 10^9^/L	100–300 × 10^9^/L

2023–2–8	14 weeks and 3 days	Routine analysis of blood	Total white blood cells: 7.10 × 10^9^/L	4–10 × 10^9^/L
			Hemoglobin: 111 g/L	110–160 g/L
			Blood platelet: 318 × 10^9^/L	100–300 × 10^9^/L

2023–2–11	14 weeks and 6 days	Routine analysis of blood	Total white blood cells: 9.50 × 10^9^/L	4–10 × 10^9^/L
			Hemoglobin: 119 g/L	110–160 g/L
			Blood platelet: 336 × 10^9^/L	100–300 × 10^9^/L

2023–2–14	15 weeks and 2 days	Routine analysis of blood	Total white blood cells: 8.50 × 10^9^/L	4–10 × 10^9^/L
			Hemoglobin: 115 g/L	110–160 g/L
			Blood platelet: 339 × 10^9^/L	100–300 × 10^9^/L

2023–2–19	16 weeks	Routine analysis of blood	Total white blood cells: 8.10 × 10^9^/L	4–10 × 10^9^/L
			Hemoglobin: 121 g/L	110–160 g/L
			Blood platelet: 356 × 10^9^/L	100–300 × 10^9^/L

2023–2–25	16 weeks and 6 days	Routine analysis of blood	Total white blood cells: 9.00 × 10^9^/L	4–10 × 10^9^/L
			Hemoglobin: 125 g/L	110–160 g/L
			Blood platelet: 349 × 10^9^/L	100–300 × 10^9^/L

2023–4–9	23 weeks	Routine analysis of blood	Total white blood cells: 11.70 × 10^9^/L	4–10 × 10^9^/L
			Hemoglobin: 123 g/L	113–151 g/L
			Blood platelet: 291 × 10^9^/L	100–300 × 10^9^/L

2023–4–18	24 weeks and 2 days	Routine analysis of blood	Total white blood cells: 8.60 × 10^9^/L	4–10 × 10^9^/L
			Hemoglobin: 121 g/L	110–160 g/L
			Blood platelet: 287 × 10^9^/L	100–300 × 10^9^/L

2023–7–24	38 weeks and 3 days	Routine analysis of blood	Total white blood cells: 10.90 × 10^9^/L	4–10 × 10^9^/L
			Hemoglobin: 133 g/L	113–151 g/L
			Blood platelet: 236 × 10^9^/L	125–350 × 10^9^/L

2023–7–28	39 weeks	Routine analysis of blood	Total white blood cells: 9.60 × 10^9^/L	3.5–9.5 × 10^9^/L
			Hemoglobin: 123 g/L	115–150 g/L
			Blood platelet: 224 × 10^9^/L	125–350 × 10^9^/L

**TABLE 2:** Timeline and results of laboratory tests (Coagulation test).

Test time	Gestational age (weeks)	Test item	Test result	Reference value
2023–1–28	12 weeks and 6 days	Coagulation tests	Prothrombin time test: 12.30 s	11–14 s
			International normalized ratio: 0.98	0.76–1.15
			Activation of thrombinogen time determination: 32.70 s	25–35 s
			Fibrinogen: 3.01 g/L	2–4 g/L
			Thrombin time: 12.80 s	14–21 s
			D‐dimer: 0.40 μg/mL	0.00–1.00 μg/mL

2023–2–8	14 weeks and 3 days	Coagulation tests	Prothrombin time test: 12.60 s	11–14 s
			International normalized ratio: 1.01	0.76–1.15
			Activation of thrombinogen time determination: 28.40 s	25–35 s
			Fibrinogen: 3.14 g/L	2–4 g/L
			Thrombin time: 13.20 s	14–21 s
			D‐dimer: 0.36 μg/mL	0.00–1.00 μg/mL

2023–2–11	14 weeks and 6 days	Coagulation tests	Prothrombin time test: 12.10 s	11–14 s
			International normalized ratio: 0.97	0.76–1.15
			Activation of thrombinogen time determination: 30.50 s	25–35 s
			Fibrinogen: 2.53 g/L	2–4 g/L
			Thrombin time: 14.90 s	14–21 s
			D‐dimer: < 0.10 μg/mL	0.00–1.00 μg/mL

2023–2–14	15 weeks and 2 days	Coagulation tests	Prothrombin time test: 13.50 s	11–14 s
			International normalized ratio: 1.08	0.76–1.15
			Activation of thrombinogen time determination: 28.80 s	25–35 s
			Fibrinogen: 3.99 g/L	2–4 g/L
			Thrombin time: 13.40 s	14–21 s
			D‐dimer: 0.40 μg/mL	0.00–1.00 μg/mL

2023–2–19	16 weeks	Coagulation tests	Prothrombin time test: 13.90 s	11–14 s
			International normalized ratio: 1.12	0.76–1.15
			Activation of thrombinogen time determination: 32.00 s	25–35 s
			Fibrinogen: 2.83 g/L	2–4 g/L
			Thrombin time: 13.20 s	14–21 s
			D‐dimer: 1.54 μg/mL	0.00–1.00 μg/mL

2023–2–25	16 weeks and 6 days	Coagulation tests	Prothrombin time test: 12.10 s	11–14 s
			International normalized ratio: 0.97	0.76–1.15
			Activation of thrombinogen time determination: 33.50 s	25–35 s
			Fibrinogen: 2.72 g/L	2–4 g/L
			Thrombin time: 13.30 s	14–21 s
			D‐dimer: 1.03 μg/mL	0.00–1.00 μg/mL

2023–4–9	23 weeks	Coagulation tests	Prothrombin time test: 10.00 s	9.6–14 s
			International normalized ratio: 0.86	0.8–1.25
			Activation of thrombinogen time determination: 24.30 s	23–38 s
			Fibrinogen: 3.88 g/L	2–4 g/L
			Thrombin time: 16.40 s	14–21 s
			D‐dimer: 1.16 μg/mL	0.00–0.50 μg/mL

2023–4–18	24 weeks and 2 days	Coagulation tests	Prothrombin time test: 11.80 s	11–14 s
			International normalized ratio: 0.94	0.76–1.15
			Activation of thrombinogen time determination: 29.70 s	25–35 s
			Fibrinogen: 3.25 g/L	2–4 g/L
			Thrombin time: 12.00 s	14–21 s
			D‐dimer: 3.73 μg/mL	0.00–1.00 μg/mL

2023–7–24	38 weeks and 3 days	Coagulation tests	Prothrombin time test: 11.20 s	9.20–15.00 s
			International normalized ratio: 0.97	0.80–1.25
			Activation of thrombinogen time determination: 23.20 s	21–37 s
			Fibrinogen: 4.94 g/L	2–4 g/L
			Thrombin time: 14.20 s	10–20 s
			D‐dimer: 3.19 μg/mL	≤ 0.55 μg/mL

2023–7–28	39 weeks	Coagulation tests	Prothrombin time test: 11.40 s	9.2–15 s
			International normalized ratio: 0.99	0.80–1.25
			Activation of thrombinogen time determination: 24.20 s	21–37 s
			Fibrinogen: 4.20 g/L	2–4 g/L
			Thrombin time: 14.80 s	10–20 s
			D‐dimer: 3.92 μg/mL	≤ 0.55 μg/mL

**TABLE 3:** Timeline and results of laboratory tests (Infection marker test).

Test time	Gestational age (weeks)	Test item	Test result	Reference value
2023–1–28	12 weeks and 6 days	Procalcitonin	0.10 ng/mL	0–0.50 ng/mL
		C‐reactive protein	4.46 mg/L	< 10 mg/L

**TABLE 4:** Timeline and results of ultrasound findings.

Test time	Gestational age	Test item	Test result	Clinical symptoms
2023–1–28	12 weeks and 6 days	Color Doppler ultrasound	Biparietal diameter: 20 mm	A small amount of dark red vaginal bleeding, without abdominal pain
			Size of hematoma: 109 mm × 41 mm	

2023–1–30	13 weeks and 1 day	Color Doppler ultrasound	Biparietal diameter: 22 mm	A small amount of dark red vaginal bleeding accompanied by mild lower abdominal pain
			Size of hematoma: 100 mm × 23 mm	

2023–2–4	13 weeks and 6 days	Color Doppler ultrasound	Biparietal diameter: 25 mm	Without abdominal pain or vaginal bleeding
			Size of hematoma: 105 mm × 17 mm	

2023–2–11	14 weeks and 6 days	Color Doppler ultrasound	Biparietal diameter: 28 mm	A small amount of dark red vaginal bleeding, without abdominal pain
			Size of hematoma: 103 mm × 27 mm	

2023–2–15	15 weeks and 3 days	Color Doppler ultrasound	Biparietal diameter: 30 mm	A small amount of dark red vaginal bleeding, without abdominal pain
			Size of hematoma: 106 mm × 23 mm	

2023–2–17	15 weeks and 5 days	Color Doppler ultrasound	Biparietal diameter: 33 mm	Without abdominal pain or vaginal bleeding
			Size of hematoma: 103 mm × 27 mm	

2023–2–24	16 weeks and 5 days	Color Doppler ultrasound	Biparietal diameter: 37 mm	Without abdominal pain or vaginal bleeding
			Size of hematoma: 88 mm × 29 mm	

2023–4–9	23 weeks	Color Doppler ultrasound	Biparietal diameter: 55 mm	Without abdominal pain or vaginal bleeding
			Size of hematoma: 55 mm × 13 mm	

2023–5–17	28 weeks and 3 days	Color Doppler ultrasound	Biparietal diameter: 72 mm	Without abdominal pain or vaginal bleeding
			Size of hematoma: 0 mm × 0 mm	

2023–6–11	32 weeks	Color Doppler ultrasound	Biparietal diameter: 81 mm	Without abdominal pain or vaginal bleeding
			Size of hematoma: 0 mm × 0 mm	

2023–7–7	36 weeks	Color Doppler ultrasound	Biparietal diameter: 83 mm	Without abdominal pain or vaginal bleeding
			Size of hematoma: 0 mm × 0 mm	

2023–7–17	37 weeks and 3 days	Color Doppler ultrasound	Biparietal diameter: 88 mm	Without abdominal pain or vaginal bleeding
			Size of hematoma: 0 mm × 0 mm	

2023–7–28	39 weeks	Color Doppler ultrasound	Biparietal diameter: 91 mm	Without abdominal pain or vaginal bleeding
			Size of hematoma: 0 mm × 0 mm	

**FIGURE 2:**
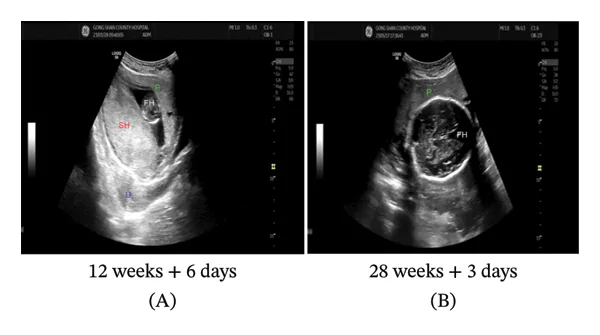
Comparison of ultrasound images before and after treatment. (A) Ultrasound images obtained at the initial diagnosis at 12 weeks and 6 days of gestation. A well‐demarcated, hypoechoic zone measuring 109 × 41 mm^2^, representing the largest cross‐sectional area, was identified beneath the gestational sac chorionic plate. Fetal examination is normal. (B) Ultrasound image at 28 weeks and 3 days of gestation after treatment. Subchorionic placental hematoma has resolved, placental echogenicity is normal, with no residual significant retroplacental or preplacental fluid collection detected, and both the fetus and amniotic fluid are normal. P represents the placenta; SH represents subchorionic hematoma; U represents the uterus; FH represents the fetal head.

Following 3 months of active fetal surveillance and supportive obstetric management, the patient’s vaginal bleeding resolved. Serial laboratory assessments, including coagulation parameters, hemoglobin concentration, and white blood cell count, remained within normal reference ranges throughout follow‐up. The SCH demonstrated progressive reduction in size and resolved completely by 28 weeks and 3 days’ gestation (Figure 2A,B). No pregnancy‐related complications were observed. Fetal growth and anatomical development remained appropriate for gestational age on serial transabdominal color Doppler ultrasonography.

A viable female neonate was delivered by repeat cesarean delivery at 39 weeks and 1 day’s gestation, indicated solely by prior cesarean delivery. The Apgar scores were 10 at 1 min and 10 at 5 min. Gross placental examination revealed normal morphology with no evidence of hematoma, retroplacental clot, or other gross pathological abnormalities. Postpartum recovery was uneventful, and no maternal complications occurred during the immediate postpartum period.

## 3. Discussion

SCH is most prevalent among individuals aged 18–30 years and represents a leading cause of vaginal bleeding during early pregnancy. A range of maternal and obstetric factors have been associated with an increased risk of SCH development. Notably, maternal hypertension is consistently identified as an independent risk factor for SCH [7]. Additionally, multiple gestation is both epidemiologically associated with higher SCH incidence and independently recognized as a significant risk factor for its occurrence [8]. In vitro fertilization (IVF) has also been associated with an elevated risk of SCH. Specifically, frozen embryo transfer (FET) (odds ratio [OR], 6.18; 95% confidence interval [CI], 1.7–22.4) and blastocyst‐stage embryo transfer (OR, 3.75; 95% CI, 1.1–13.3) are independently identified as significant risk factors for SCH [9]. In addition, coagulation dysfunction, abnormal immune function, infection, and abnormal oxidative stress are also considered as risk factors for SCH [6]. In contrast, anterior placental location is associated with a reduced risk of SCH and is considered a potential protective factor [10]. Notably, no statistically significant associations were observed between SCH incidence and maternal age, self‐reported race, body mass index (BMI), or prior abortion history [11]. In this case, the patient has no history of hypertension, diabetes, chronic kidney disease, autoimmune disorders, pelvic inflammatory disease, systemic vascular disease, coagulation disorders, recurrent miscarriage, or abdominal trauma. This is a spontaneous pregnancy with no high‐risk factors for SCH, which is a rare case in the occurrence of SCH and is worthy of further study. This underscores the clinical reality that even in the absence of typical predisposing conditions, severe SCH can occur, highlighting the need for increased vigilance and prompt intervention.

According to the current obstetric guidelines, the first trimester is defined as gestation prior to 14 weeks and the second trimester extends from 14 to 28 weeks. In this case, the diagnosis was established at the latest stage of the first trimester, and the vast majority of the clinical management, follow‐up, and resolution of the hematoma occurred solidly within the second trimester. Given the significant differences in the clinical approach and prognosis for SCH between the first and second trimesters, and to accurately reflect the clinical course where the key interventions and observations took place, we have retained the classification as a second‐trimester case.

SCHs identified in the first and second trimesters of pregnancy demonstrate significant differences in their prognostic profiles [8, 12]. Early‐stage SCH primarily increases the risk of miscarriage, while SCH occurring in the second and third trimesters is more likely to induce placenta‐related complications. The core risk of SCH in early pregnancy (especially before 12 gestational weeks) is pregnancy loss [13]. Clinical studies have shown that the earlier the gestational week at which SCH occurs, the higher the risk of miscarriage: the pregnancy failure rate can reach 19.6% when SCH occurs before 7 gestational weeks [12, 14, 15]. However, the association between first‐trimester SCH and miscarriage remains controversial, and some scholars argue that SCH does not increase the risk of miscarriage [11, 16, 17]. Furthermore, some literature suggests that early SCH is not associated with adverse pregnancy outcomes in the second and third trimesters [18, 19], but existing literature also indicates that early SCH still increases the risk of preterm delivery, placental abruption, and stillbirth [20]. After entering the second trimester of pregnancy, the risk of miscarriage decreases significantly, while the risk of placenta‐related complications increases remarkably. Recent research shows that SCH identified between 12 and 20 gestational weeks is more likely to cause preterm delivery than that identified before 12 gestational weeks [10]. Late‐onset SCH is more prone to trigger severe obstetric emergencies, including placenta previa, placental abruption, and placental adhesion [21]. Nevertheless, most existing relevant studies are small‐sample investigations with inconsistent results. Therefore, the prognostic difference between first‐trimester and second‐trimester SCH still requires further investigation via large‐sample studies. In general, the gestational week‐specificity of SCH determines its risk stratification. In clinical practice, a single management mode should be abandoned: The core management focus should be miscarriage prevention in the first trimester, while the primary goal should be prevention of placenta‐derived complications in the second and third trimesters. Moreover, individualized dynamic monitoring should be implemented based on the size of the hematoma and the status of the mother and fetus.

Transabdominal and transvaginal ultrasound remain the primary imaging modalities for diagnosing SCH. There is still no unified standard internationally or domestically for assessing the severity of SCH. In clinical practice, semiquantitative grading is most commonly performed using either the area ratio (hematoma area relative to gestational sac area) or the volume ratio (hematoma volume relative to gestational sac volume). The area‐ratio classification stratifies SCH as mild (< 33%), moderate (33%–50%), or severe (> 50%); the volume‐ratio method employs a four‐tier system: < 10%, 10%–25%, 25%–50%, and > 50% [6, 12, 22]. In this case, quantitative ultrasound measurement demonstrated an area ratio exceeding 50%, confirming the diagnosis of a massive SCH. Notably, as a rare subtype of SCH, massive SCH is associated with significantly elevated risks of adverse pregnancy outcomes compared with small SCH, including preterm birth, spontaneous abortion, intrauterine growth restriction, and first‐trimester vaginal bleeding [23–25]. Furthermore, SCH size demonstrates a positive correlation with several pregnancy‐related complications, including gestational hypothyroidism, intrahepatic cholestasis of pregnancy, gestational hypertensive disorders, term premature rupture of membranes, and gestational thrombocytopenia [26]. A prospective cohort study further reported that patients with medium or massive SCH exhibited earlier gestational age at delivery relative to those without SCH [10]. However, existing studies on massive SCH remain limited by small sample sizes, and consensus on the precise definition of “massive SCH” has yet to be established. Consequently, elucidating the clinical implications of massive SCH remains a critical research priority. Moreover, several reports have questioned the prognostic relevance of SCH size or maximal diameter, suggesting it may not be an independent predictor of adverse perinatal outcomes [8]. Thus, the association between SCH size and perinatal prognosis remains inconclusive and warrants further validation through large‐scale, multicenter prospective studies.

There are no guidelines for the management of massive SCH. Given the associated risk of secondary infection of hematoma and subsequent preterm premature rupture of membranes, the antibiotic was used specifically to prevent these potential infectious complications [6]. However, this was only a clinical decision tailored to this specific high‐risk case and was not a universally recommended strategy, and further prospective studies were needed to validate its role. Considering that blood stimulation might induce uterine contractions, intramuscular progesterone administration was performed to suppress uterine contractions [6]. In addition, regular monitoring of biochemical indicators such as hemoglobin, coagulation function, C‐reactive protein, and hematoma status was performed. Although the present case has achieved a successful outcome, as a single case report, its therapeutic efficacy still requires validation via subsequent large‐sample, multicenter clinical data. Moreover, antioxidant therapy with *α*‐lipoic acid holds promise as a novel therapeutic strategy for SCH. Current evidence suggests that pregnant individuals treated with alpha‐lipoic acid experience a significantly lower spontaneous abortion rate relative to those receiving vaginal progesterone [27–29]. Immunomodulatory therapy remains investigational. Preliminary clinical data indicate that intravenous porcine immunoglobulin administered in combination with dydrogesterone may promote hematoma resolution and improve perinatal outcomes [30]. Additionally, certain traditional Chinese medicine formulations, including ANTAI YIN and ZISHEN YUTAI WAN, have demonstrated encouraging clinical efficacy in SCH management [31]. However, robust validation through well‐designed randomized controlled trials is still lacking. Collectively, these interventions represent emerging avenues for SCH treatment, and their integration into routine clinical practice awaits confirmation from high‐quality prospective studies.

However, the present study has certain limitations. Due to the hospital’s lack of magnetic resonance imaging (MRI) equipment, MRI‐assisted assessment of placental lesions could not be performed; meanwhile, pathological examination of the placenta was not conducted after delivery. The absence of the aforementioned imaging and histological evidence made it impossible to definitively exclude or confirm the diagnosis of Breus’ mole in this case either antepartum or postpartum. Such assessments should be integrated into the clinical management of SCH to enhance diagnostic accuracy for this rare condition. Moreover, we recognize that the 20‐mg/day intramuscular progesterone dose used in this study is insufficient. In the future, higher intramuscular dosages of 40–100 mg/day will be adopted to achieve better therapeutic effects.

In conclusion, this study provides a detailed, real‐world example of managing a high‐risk, massive SCH in the absence of standardized guidelines, which provides a practical foundation for future research.

## Funding

The study was supported by Special Fund for People’s Livelihood Research of Pudong New Area Science and Technology Development Fund (Grant No. PKJ2024‐Y50) and the Key Subject Group Of Pudong New Area Health System In Shanghai (Grant No. PWZxq2022‐15).

## Ethics Statement

Gongshan People’s Hospital Ethics Committee had approved the research project protocol, and it conformed to the provisions of the Declaration of Helsinki in 1995. Written informed consent was obtained from the patient involved in the study, including the use of images and medical records for publication.

## Conflicts of Interest

The authors declare no conflicts of interest.

## Data Availability

All the data in the research are included in this manuscript.
